# EXAMINING THE PRELIMINARY EFFICACY OF AN ONLINE ASYNCHRONOUS PSYCHOEDUCATION COURSE FOR BLACK DEMENTIA CAREGIVERS

**DOI:** 10.1093/geroni/igad104.2751

**Published:** 2023-12-21

**Authors:** Sloan Oliver, Karah Alexander, Nkosi Cave, Fayron Epps

**Affiliations:** Emory University, Atlanta, Georgia, United States; Emory University, Atlanta, Georgia, United States; Emory University, Atlanta, Georgia, United States; Emory University, Atlanta, Georgia, United States

## Abstract

Caregiving While Black (CWB) is an online asynchronous psychoeducation course designed to facilitate the growth of caregiver mastery in Black caregivers of persons living with dementia (PLWD), while increasing the PLWD’s quality of life and providing resources. CWB was co-developed by Black caregivers, PLWD, and healthcare professionals who identified the need for a responsive, culturally tailored course to combat specific burdens faced by Black caregivers. Course topics included navigating the healthcare system, managing home, and self-care competencies. This pilot study tested the preliminary efficacy of CWB, which included 8-10 hours of content delivered in 37 short interactive segments. Thirty-two Black caregivers completed the course over a 10-week time frame. Socio-demographic information and measures of psychological well-being and self-efficacy were collected at baseline, midpoint, and end of study. Data were analyzed using descriptive statistics and t-tests. Results confirmed the course’s ability to improve caregivers’ emotional well-being and sense of confidence and mastery in their caregiving role. Pre- and post-course data revealed a significant improvement in caregiver depression (p=.009), burden (p=.034), and role strain (p=.029) within 30 days of course completion. All statistically significant improvements had moderate effect sizes > 0.38 (Cohen’s d). The frequency of behavioral problems in PLWD decreased (p=.033), as did caregiver reactions to those behaviors (p=.0054). Further, caregiver mastery from baseline to course completion increased 0.42 points, on average, with an effect size of 0.26 (Cohen’s d). These results exemplify the need for novel, culturally tailored and responsive courses like CWB.

